# Preparation and tribological properties of multi-layer graphene/silicon dioxide composites-based solid lubricant coatings at elevated temperatures

**DOI:** 10.1098/rsos.220740

**Published:** 2023-02-08

**Authors:** Wei Wang, Wenjuan Chang, Shijie Ding, Yishen Qu, Yuan Gao, Kuaishe Wang

**Affiliations:** ^1^ School of Metallurgy Engineering, Xi'an University of Architecture and Technology, Xi'an 710055, People's Republic of China; ^2^ School of Economics and Management, Beijing Jiaotong University, Beijing, 100044, People's Republic of China

**Keywords:** sol–gel method, MLG/SiO_2_ composite, solid lubricant coatings, high-temperature tribological properties, lubrication mechanisms

## Abstract

The solid lubricating coatings have an important role in hot metal forming. However, traditional lubricants cannot be applied to the harsh working conditions. In this investigation, the novel solid lubricant coatings including multi-layer graphene (MLG)/silicon dioxide (SiO_2_) composites and sodium metaphosphate phosphate were prepared. The high-temperature tribological properties of the solid lubricant coatings were investigated by friction and wear tester. The experimental results showed that SiO_2_ nanoparticles were evenly grafted by sol–gel method on the surface of MLG, forming MLG/SiO_2_ composites. MLG/SiO_2_ composites presented excellent thermal stability at 800°C. In the range of 400–800°C, the average coefficients of friction (COFs) were decreased from 0.3936 to 0.3663, and then increased from 0.3663 to 0.4226. Based on the analysis of wear scar, the lubrication mechanisms of the solid lubricating coatings were proposed. The low interlayer shear of MLG and the ball bearing of SiO_2_ nanoparticles are the main reason for the reduction of COFs. In addition, the tribo-chemical reaction film formed on the frictional interface could protect the contact surfaces from severe damage. The findings would be beneficial for developing novel lubricants for hot metal forming process.

## 1. Introduction

Metallic materials are extensively used in military industry, aerospace, machinery manufacturing and other fields. Metal forming technologies such as rolling, forging and extruding are the key to achieve different applications. In the process of metal forming, the designed shapes, surface quality and mechanical properties of products are always affected by the friction and wear [1,2]. To reduce wear and improve the surface quality of the products, many liquid and solid lubricants are used in the fields of the metal processing [3]. For hot metal forming processes, the temperature of the metal forming is always over 350°C, traditional liquid lubricants, e.g. water-based lubricants, oil-based lubricants cannot sustain extreme high-temperature conditions due to their low ignition point and combustion [4,5]. Therefore, solid lubricants which reduce friction in harsh working conditions are widely used in hot metal forming processes [6–9].

There are many kinds of high-temperature solid lubricants such as metal oxides, metal sulfides, inorganic borates and rare earth compounds [10,11]. However, most of the solid lubricants are based on the layered structure materials with weak interlayer forces, for instance, graphite, molybdenum disulfide (MoS_2_), polytetrafluoroethylene (PTFE), h-BN, etc. Arsan *et al*. [12] investigated the tribological behaviour of MoS_2_/Nb coatings from room temperatures up to 500°C. The applied optimal temperature and the lowest coefficient of friction (COF) value are 100°C and 0.014, respectively. As the temperature increased, the oxidation rate of the coatings and COFs are increased rapidly. Kim *et al*. [13] investigated tribological behaviour of graphite used as sealing materials at room and elevated temperature. It was found that COF at 485°C is lower than that at room temperature under same experimental conditions. Conventional solid lubricants with layered structures such as graphite and MoS_2_ can meet the requirements of low-temperature operation, but they can be easily oxidized at high temperatures [14]. In recent years, many scholars have carried out extensive research on improving the tribological properties of high-temperature solid lubricants. The selected lubrication materials are needed for improving their oxide resistance and lubricating properties to achieve their full potential. The key to the coating design is to meet the requirements of the wear resistance, withstanding high temperatures and loading forces [15,16]. Up to now, there are still huge problems in the application of the high-temperature solid lubricants.

In recent years, the lubrication of composite has attracted more and more attention, due to the combination of two or three fillers to improve the tribological properties of composites [17–19]. Multi-layer graphene (MLG) is the hexagonal honeycomb structure consisting of the layers of carbon atoms, which has various potential applications, such as nanoelectronics, sensors, solar cells and composites, due to its excellent properties [20,21]. MLG as a two-dimensional material has an ultra-thin layer structure and very low interlayer shear strength. It has the characteristics of low surface energy and atomically smooth surface, which help the sliding between the layers to reduce COF and prevent direct contact with the friction specimens [22–24]. For high-temperature and high-pressure environments, MLG has the fatal weakness of being prone to oxidation at higher temperatures. Thermal decomposition of MLG occurs at 600°C. As the temperature is increased, the complete decomposition to form CO_2_ will happen [25,26]. MLG exhibits relatively poor stability because it is easy to be oxidized at high temperature. These defects were improved by grafting amorphous silicon dioxide (SiO_2_) nanoparticles on the surface of MLG [27]. Nanometer SiO_2_ is a kind of ultra-fine material, has a large specific surface area, is non-toxic and tasteless, has high-temperature resistance, high strength and high toughness and other excellent properties [28,29]. SiO_2_ nanoparticles in composites can improve the solidity of solid lubricant coatings and carry severe rubbing stress [30]. Due to the low oxygen permeability of SiO_2_, when SiO_2_ is attached to the surface of MLG, it can slow down the diffusion of oxygen, thus reducing the oxidation of graphene [31,32]. Koh *et al*. [27] investigated whether depositing SiO_2_ layer on the carbon surface would improve the oxidation resistance of carbon. The results showed that the oxidation resistance of carbon increased by a factor of 5 with the delayed passage of oxygen through the SiO_2_ layer. Therefore, the MLG/SiO_2_ composites as the solid lubricant coatings could effectively reduce the oxidation of MLG at the high temperature and present excellent lubricating properties. However, the research for tribological properties and oxide resistance of the MLG/SiO_2_ composites as the solid lubricant coatings at elevated temperature has still been insufficient.

In this investigation, a new type of high-temperature solid lubricant coatings based on MLG/SiO_2_ composites has been developed. The present study concerns the tribological properties of MLG/SiO_2_ composites-based solid lubricant coatings with limits of temperatures from 400°C to 800°C. It provides theoretical basis for high-temperature metal plastic forming lubrication.

## 2. Experiment

### 2.1. Materials

In the experiments, MLG (Pioneer Nano), deionized water, anhydrous ethanol (analytical grade), ammonia (NH_3_ · H_2_O), ethyl orthosilicate (TEOS, Sinopharm) and sodium metaphosphate (NaPO_3_, Sinopharm) were used as the main experimental materials. All chemicals and reagents were used without further purification.

### 2.2. Preparation of multi-layer graphene/silicon dioxide composites

**Figure 1.**
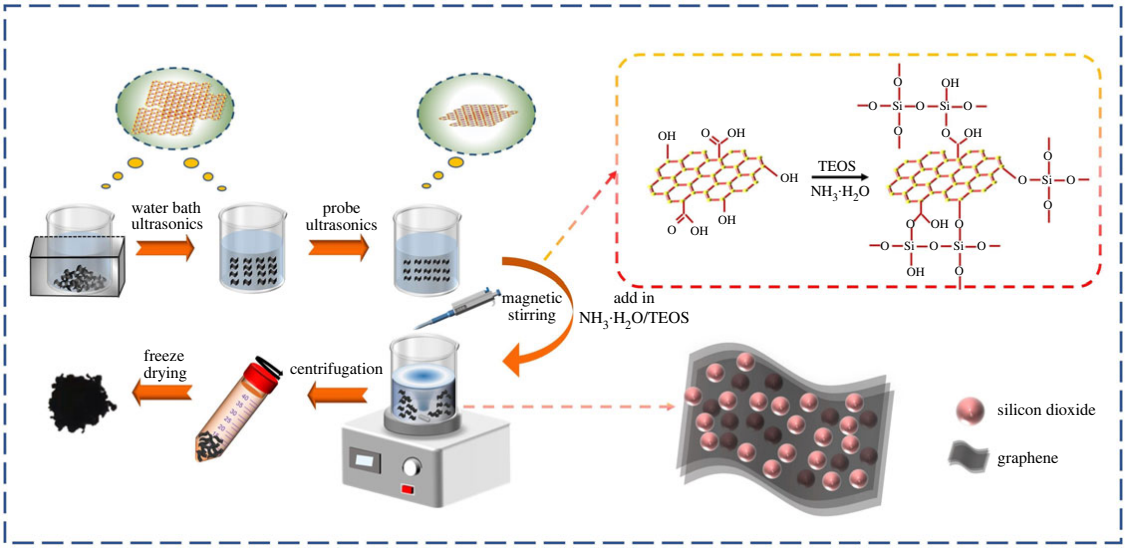
The scheme of preparation process of MLG/SiO_2_ composites.

The scheme of preparation process of MLG/SiO_2_ composites is shown in figure 1. The MLG/SiO_2_ composites were prepared by sol–gel method. The preparation process of the composites was as follows. A 0.1 g of MLG powders were dispersed into the mixture of 7 ml of the deionized water and 29 ml of the ethanol by ultrasonication to obtain the uniformly dispersed MLG solution. The prepared MLG solution was sonicated using a horn probe sonic tip at 500 W for 3 h, further stirred by the magnetic stirrer at 25°C. During the stirring process, 2.5 ml of NH_3_ · H_2_O and 3 ml of TEOS were added drop wisely into the mixture solution [28,33]. And then, the prepared solution was centrifuged at 8000 r.p.m. for 15 min and the precipitates were collected. Finally, the precipitates were repeatedly washed with the deionized water and ethanol several times, and the MLG/SiO_2_ powders were obtained by the freeze-drying oven.

### 2.3. Preparation of the solid lubrication coatings

The MLG/SiO_2_ composite and NaPO_3_ were used as the main lubricants and binder, respectively. The homogeneous slurries of 1.2 g MLG/SiO_2_ powders, 0.15 g NaPO_3_ and 7 ml water were obtained by stirring at 100°C for 2 h. The prepared slurries were used for preparing the coatings. The substrate material is the titanium alloy (Ti6Al4V) with a radius of 25.4 mm and a thickness of 6 mm. Prior to coating, the discs were cleaned by ultrasonic cleaning process to remove contaminants. The coatings were fabricated by the spin processor on the surface of titanium alloy, and the optimal thickness was 40 µm after several tests. The prepared discs coated with solid lubricant were used in subsequent friction tests.

### 2.4. Oxidation resistance at high temperature

In this investigation, the MLG/SiO_2_ composites were heat treated to evaluate the oxidation resistance properties at high temperature. Six equal parts of the composite powders were prepared and heated with the rate of 8°C min ^−1^ from room temperature to various target temperatures (400°C, 500°C, 600°C, 700°C and 800°C) for 1 h by tube furnace (gxl-1600) in an air atmosphere [34].

### 2.5. Tribological testing

The ball-on-disc tests were carried out by Bruker UMT-TriboLab Machine at multiple target temperatures. The upper specimen for the frictional test is the silicon nitride (Si_3_N_4_) ball with a diameter of 10 mm, and the lower specimen is the Ti6Al4V titanium alloy disc with the solid lubricant coatings. The specimens were assembled onto the friction testing machine and heated from room temperature to target temperatures of 400°C, 500°C, 600°C, 700°C and 800°C for 30 min. In the frictional experiments, the load, rotating rate and radius are 50 N, 62.83 mm s^–1^ and 20 mm, respectively. All tests were repeated three times under the same condition to ensure the reproducibility of the results. For ball–disc contact, an elliptical contact zone is generated at the contact point under external load [35]. The corresponding Hertzian stress is calculated by Hertz's theory. The Hertz contact stress (*p*) and contact diameter (*a*) were calculated using equations (2.1) and (2.2), where W is applied load, *R* is ball radius, and *E*′ (183 GPa) is effective elastic modulus, which can be calculated by equation (2.3). In the equation, *E*_1_ (319 Gpa) and *E*_2_ (110 GPa) are the elastic modulus of the friction pairs, and *μ*_1_ (0.28) and *μ*_2_ (0.34) are the Poisson ratios [36]. In this study, the corresponding contact stress is 2.086 GPa by formula.

2.1 $$p=\frac{4W}{\pi a^{2}},$$

2.2 $$a=2{\left(\frac{2}{3}\times \frac{WR}{E^{\prime }}\right)}^{1/3}$$

2.3 $$\mathrm{and}\;\;\frac{1}{E^{\prime }}=\frac{1}{2}\left(\frac{1-\mu_{1}^{2}}{E_{1}}+\frac{1-\mu_{2}^{2}}{E_{2}}\right).$$

The wear rate of the Si_3_N_4_ balls was calculated by the following formulae, where V is the wear volume, *h* is the depth of wear track, *r* is the radius of the ball, *b* is the width of wear surface, *K* is the specific wear rate, W is the applied load on the ball and S is the sliding distance.

2.4 $$V\;=\frac{2\pi hr(3h^{2}+4b^{2})}{6b}$$

and

2.5 $$K=\frac{V}{W\cdot S}.$$

### 2.6. Characterization

The MLG/SiO_2_ powders were examined by X-ray diffractometry (XRD, D8 Advance A25) with scanning from 10° to 80° (2*θ*) and using a step size of 8° min^−1^. The morphologies of the MLG and MLG/SiO_2_ powders and the elemental distribution of the powders were analysed by the Gemini SEM 300 equipped with an energy-dispersive X-ray spectrometer (EDS). Atomic force microscopy (Bruker Multimode 8) was used to examine the size of the MLG. The heat-treated composite powders were analysed by XRD with scanning from 10° to 80° (2*θ*) and the step size of 8° min^−1^ to determine whether phase transformation occurred. And the TG-FTIR machine consisting of the thermogravimetric analyser (iS50 TGA/DSC3) and the Fourier transform infrared spectrometer (ISQ 7000 Nicolet) was used to evaluate the thermal stability of the powders. The oxidation resistance of the composite powders was evaluated by the above detection and analysis instruments.

After frictional experiments, the morphology of Si_3_N_4_ was evaluated by optical microscopy. And the wear scars of the titanium alloy were examined by scanning electron microscope (SEM) and EDS to obtain the elemental distribution. Raman spectroscopy (LabRAM HR Evolution, with laser excitation of 532 nm) was used to confirm the structural features of solid lubricants on the wear scars after tribological experiments. X-ray photoelectron spectroscopy (XPS, Thermo ESCALAB 250XI) was applied to analyse the chemical composition and elemental valence of the wear scars.

## 3. Results and discussion

### 3.1. Materials characterization

**Figure 2.**
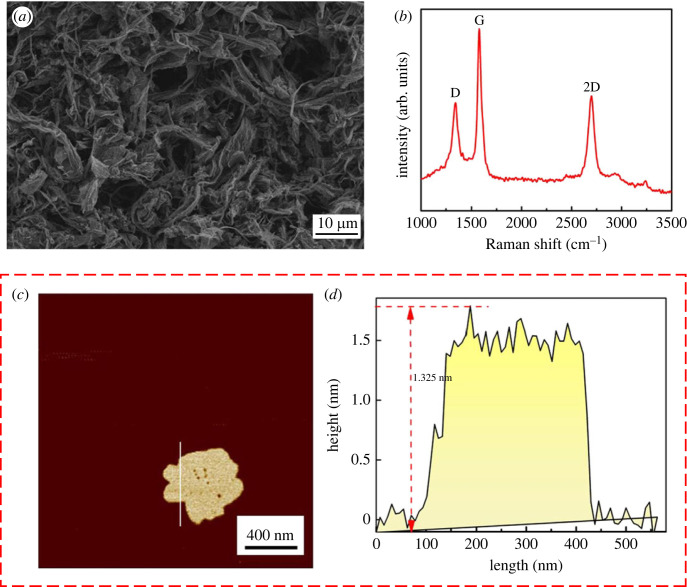
(*a,b*) SEM image and Raman spectra of MLG, and (*c,d*) atomic force microscope (AFM) image and height profile of MLG.

The morphology of MLG was examined by SEM, as shown in figure 2*a*, which showed that MLG was composed of aggregated flakes. As shown in figure 2*b*, main features in Raman spectra of MLG are D, G and two bands, which located at 1350 cm^−1^, 1580 cm^−1^ and 2700 cm^−1^, respectively [37,38]. Atomic force microscope (AFM) is considered to be an important method to characterize MLG, and provides three-dimensional images to judge the number of the layers [39]. The AFM image of MLG is shown in figure 2*c*; the height of the MLG was nearly 1.325 nm. Through the theoretical calculation, it indicated that the present MLG is about four layers.

**Figure 3.**
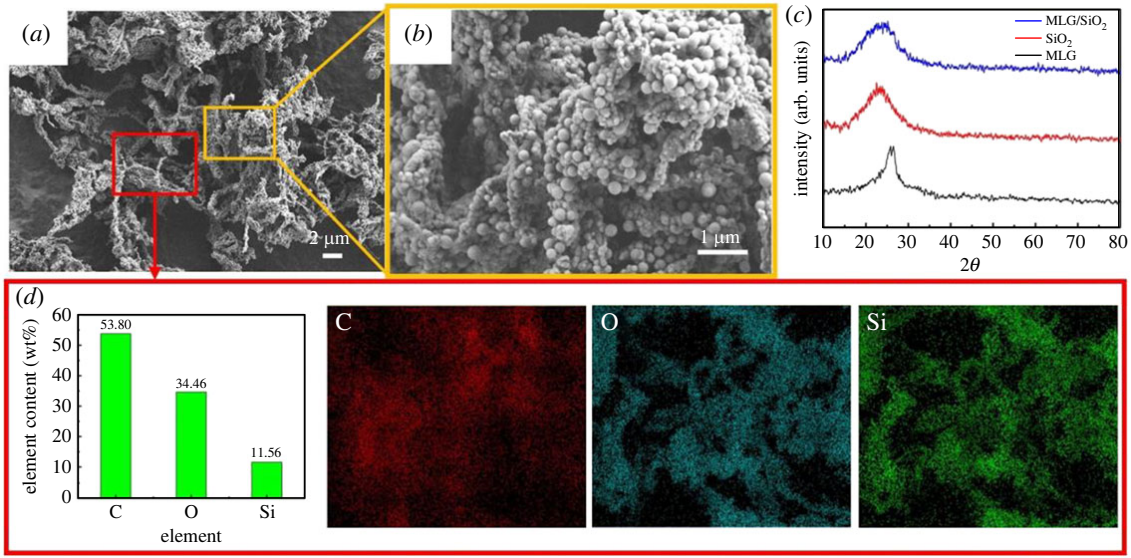
(*a,b*) SEM images of MLG/SiO_2_ powders, (*c*) XRD patterns of MLG/SiO_2_, SiO_2_, MLG powders and (*d*) EDS maps of MLG/SiO_2_ powders.

To study the microscopic morphology and phase components of the powders, SEM images and XRD patterns of MLG/SiO_2_ powders were further analysed. As shown in figure 3*a*, the SiO_2_ nanoparticles were uniformly distributed on the surface of MLG, and the overall morphology is similar to the typical two-dimensional folded structure of MLG, which retains the frictional resistance reduction property of MLG [28]. The ratio of NH_3_ · H_2_O to TEOS was adjusted to 5 : 6, and the size of the prepared SiO_2_ particles were in the range of 120–350 nm [40] (figure 3*b*). Figure 3*c* provides the XRD images of MLG/SiO_2_ composites, amorphous SiO_2_ and MLG. MLG exhibited the sharp peak at the diffraction angle (2*θ*) of 26.1°, and the broad diffraction peak of SiO_2_ was observed at 22.6°, indicating that amorphous SiO_2_ was presented. The composite powders present the clear peak of SiO_2_ in the range of 18–35°, it indicates that the distribution of SiO_2_ particles in the composite is in disorder. In addition, no characteristic peak of MLG was found, the reason is that the disordered distribution of MLG in the composite and the large number of SiO_2_ particles covered the surface of MLG [41]. These results showed that SiO_2_ and MLG were successfully compounded and that SiO_2_ particles were uniformly distributed on the surface of MLG. Figure 3*d* shows the EDS analysis of the powders in the red area in (*a*), which shows that the elements contained in the powder are C, O and Si, and their contents are 53.8%, 34.64% and 11.56%, respectively.

**Figure 4.**
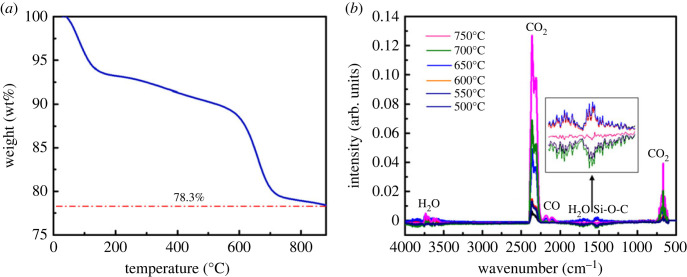
TG-FTIR analysis (*a*) TGA curve of MLG/SiO_2_ powders. (*b*) FTIR spectra of volatile gases were collected at 500°C, 550°C, 600°C, 650°C, 700°C and 750°C.

The thermal stability of MLG/SiO_2_ composites is very important for high-temperature applications. Therefore, the thermal properties of the prepared MLG/SiO_2_ powders were evaluated by TG-FTIR spectroscopy at air atmosphere (figure 4). From figure 4*a*, it shows that an obvious change was observed in the mass loss of the sample with increasing temperature. For the composite powders, there are four stages in the whole procedure. In the first stage, it was observed from the TGA curve that the mass loss before 150°C was about 7.0 wt%. It is caused by the loss of water from the composite powders. The second stage is 200–600°C; compared with the first stage, the curve is flatter and the mass loss is about 4.0 wt%. On the one hand, the mass loss is caused by the thermal decomposition of the residual TEOS in the composite and the decomposition of the organic groups in the silica. On the other hand, it is caused by the thermal decomposition of some MLG [42,43]. The third stage is 600–700°C, and the mass loss is about 9.0 wt%, which is mainly caused by the thermal decomposition of MLG. From 700°C to 800°C, it is the fourth stage, with a mass loss of about 2.0 wt%. When the powders were heated to 880°C, there are 78.0 wt% of the mass remained, indicating that the MLG/SiO_2_ composites have good thermal stability.

In order to qualitatively evaluate the loss of the composites with increasing temperature, FTIR spectra were adopted simultaneously with TGA analysis. The FTIR showed the absorption peaks of different gases at different temperatures (figure 4*b*). The peaks within 4000–3500 cm^−1^ and 2000–1580 cm^−1^ represent O-H bond stretching vibration bending peaks, indicating the existence of water. The absorption bands of Si-O-C appear where the wavenumber is 1300–1580 cm^−1^, it shows that SiO_2_ grafts on surface of MLG through Si-O-C bands [44]. The stretching vibration peaks representing C=O at 2400–2250 cm^−1^ and 800–550 cm^−1^, indicate the presence of CO_2_ gas. The stretching vibration peak at 2250–2000 cm^−1^ represents the C-O bond, indicating the presence of CO gas [1]. With the increase of temperature, the correlation peak intensity of CO_2_ was firstly increased and then decreased, the maximum value was obtained at 650°C. However, the intensity of the peak associated with H_2_O did not change greatly with increasing temperature. The weight of MLG/SiO_2_ powders is mainly lost due to the changes of water and silica before 600°C. The weight loss between 600°C and 750°C is severe, which is mainly caused by the thermal decomposition of MLG to generate carbon dioxide gas. It is shown that MLG/SiO_2_ powders can withstand high temperature of 600°C and still retains part of MLG between 700 and 880°C, which is sufficient to show that SiO_2_ has a protective effect on the oxidation of MLG.

**Figure 5.**
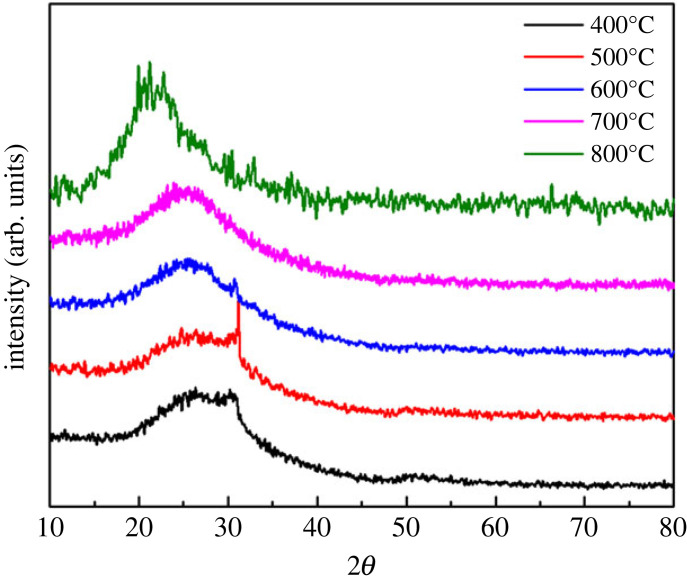
Heat-treated XRD of MLG/SiO_2_ powders: showing the heat treatment XRD curve of 400°C, 500°C, 600°C, 700°C and 800°C.

The phase analysis of MLG/SiO_2_ composite powders processed at different temperatures was carried out by XRD. As shown in figure 5, the processed powders in the temperature range of 400–700°C show the characteristic peaks of MLG/SiO_2_ powders at 2*θ* of 25°. However, the characteristic peak of the powders at 800°C was shifted compared with that at 700°C, which may be caused by the gradual transition from amorphous SiO_2_ to microcrystalline SiO_2_. From 400°C to 600°C, the obvious peaks appeared near 2*θ* of 31°. Due to the presence of residual TEOS in the composite powders, it is susceptible to pyrolysis and carbonization under high temperature easily transformed into the amorphous carbon. The carbon was collected on the surface of the composite material as a carbon layer or diffusely distributed. At 700–800°C, amorphous carbon reacts with SiO_2_ into CO gas [45–47].

3.1 $${\mathrm{SiO}}_{2}(s)+C(s)\to \mathrm{SiO}(g)+\mathrm{CO}(g).$$

### 3.2. Tribological experiment

**Figure 6.**
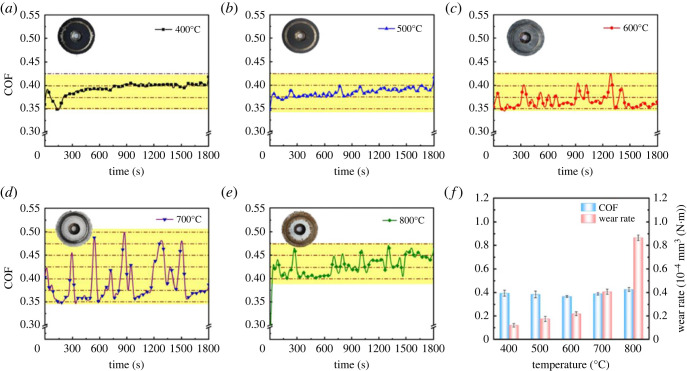
(*a–e*) The COF curves at different temperatures and (*f*) wear rate of Si_3_N_4_ balls.

The operating temperature is very important to the tribological performance. Therefore, as shown in figure 6, the tribological properties at various temperatures were investigated. It can be clearly seen that the colours of the coating at 400–500°C are black. At 600°C, the colour of the coating was changed from black to grey. The coating colour is white at 700–800°C. Figure 6*a* shows the frictional curve at 400°C, COFs were decreased until 150 s, the COFs present an increasing trend between 150 s and 300 s, and then gradually stabilize. The frictional curve at 500°C is reported in figure 6*b*; the COFs were slowly increased from 0.35 to 0.425. The average COFs of the test temperature at 400°C and 500°C were 0.3936 and 0.3845, respectively. Figure 6*c* shows the frictional curve for 600°C, the COFs were raised gradually in the beginning of 40 s, after a period of time, the COFs fluctuated around 0.35, and the average COF is 0.3663. Figure 6*d* is the frictional curve at 700°C, the COFs fluctuated greatly between the value of 0.35 and 0.50 and the average COF is 0.3894. Figure 6*e* is the frictional curve at 800°C, the minimum COF is maintained at about 0.40, and its average friction coefficient is 0.4266. From the above description, it is not difficult to find that temperature has a significant effect on the COFs of solid lubricating coating. In this investigation, the average COF at 600°C is the smallest, and it is 15% lower than that at 800°C.

As shown in figure 6*f*, as the temperature increased from 400°C to 800°C, the average COFs were firstly decreased and then increased, while the wear rates have presented an increasing trend, and it has not been consistent with the same trend of COFs. Wear and friction are complex surface-dependent processes that are affected by many factors, including material properties, roughness, friction form, load, speed, temperature, humidity, chemical reaction, loading conditions, environment and other complex factors. Temperature affects the material's wear to a large extent by changing the material's mechanical strength, adhesion and oxidation kinetics. A lot of factors affect the COFs and wear rates, a lot of experimental work is needed in the future to complete [48]. In this work, the increase of wear rate may be caused by the plastic deformation and softening of the coating and titanium alloy substrate under high temperature and high pressure [49,50]. Yu *et al*. [50] investigated the high-temperature wear resistance of S8-POSS/PAI/PTFE lubricating coating. They found that the COF of PAI/PTFE lubricated coating decreased as the temperature increased, while the wear rate increased. The wear rate increased significantly with the rise of temperature, which is mainly due to the softening and severe plastic deformation of the coatings at high temperature. The increase of wear rate is caused by softening and plastic deformation of the coating at high temperature.

### 3.3. Wear surface analysis

**Figure 7.**
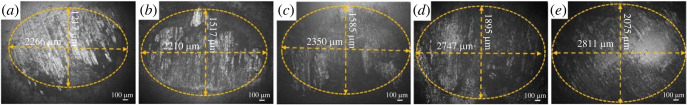
(*a–e*) The optical microscopy image of wear surface of Si_3_N_4_ balls at 400°C, 500°C, 600°C, 700°C and 800°C, respectively.

The optical microscope images at the wear surface of Si_3_N_4_ balls are shown in figure 7. The wear surfaces of the Si_3_N_4_ balls are close to an elliptical shape and the wear areas increase with the increase of temperature (figure 7*a–e*). The elliptical shape is formed due to the local deformation of the Si_3_N_4_ ball under the action of external load [51]. The wear scars of 400°C (figure 7*a*) and 500°C (figure 7*b*) are more severe compared with the wear scars of 600°C (figure 7*c*); the reason for this phenomenon was more adequate lubrication at 600°C [52]. Figure 7*d,e* shows the macroscopic morphology of the worn surface of the balls between 700°C and 800°C; it confirms that adhesive wear occurred due to the softening of the titanium alloy in the high temperature section. The wear debris of titanium alloy was transferred into the surface of the Si_3_N_4_ balls during friction and wear processes.

**Figure 8.**
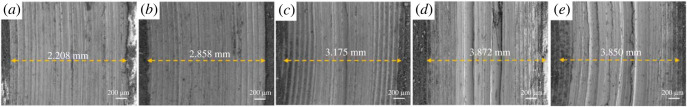
(*a–e*) The SEM image of wear scar of titanium alloy discs at 400°C, 500°C, 600°C, 700°C and 800°C, respectively.

Figure 8*a,b* is the SEM images of the wear scars at 400°C and 500°C, there are many shallow scratches and abrasive particles on the surface of titanium alloy. Figure 8*c* is an image of the wear scars at 600°C. Compared with the wear scars between 400 and 500°C, the wider wear scar appeared in the middle, while the wear surface is overall flattened and has fewer chip particles. Figure 8*d,e* are the SEM images of the wear scars at 700°C and 800°C, there are many deep wear furrows of the wear trace, but wear debris particles on the surface of titanium alloy are less, and the main reason for these phenomena is due to the softening of the titanium alloy [49].

**Figure 9.**
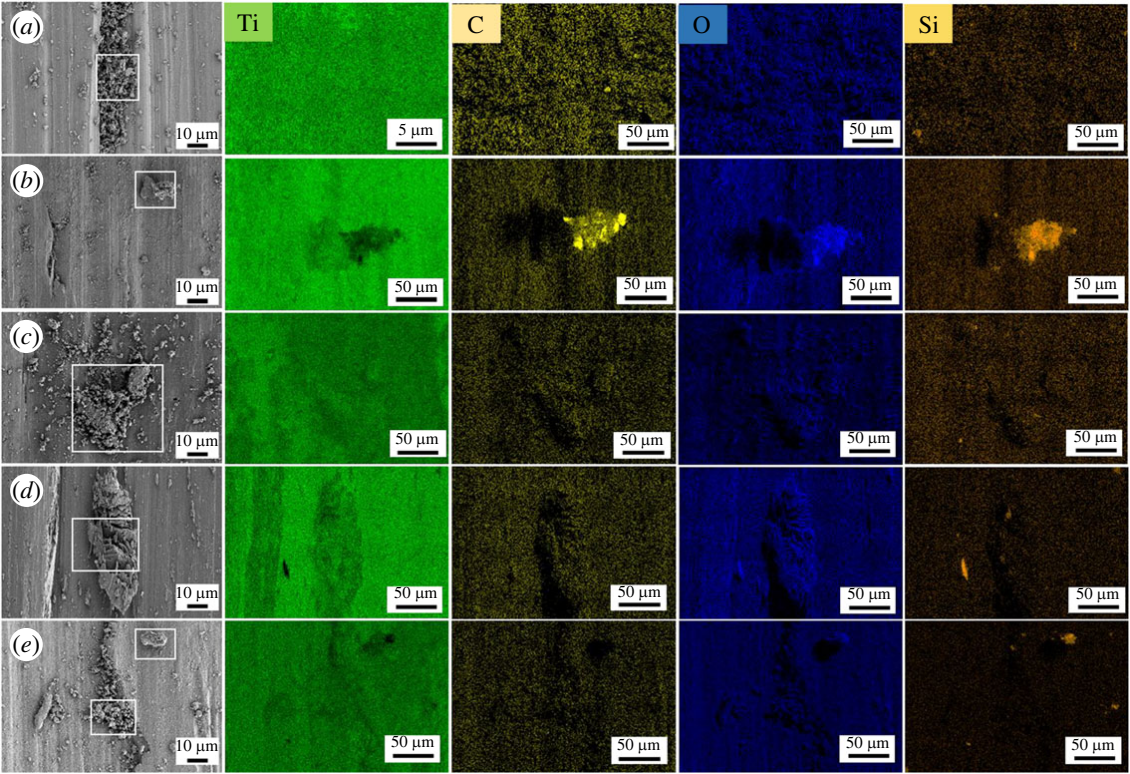
EDS images of wear scar of Ti6Al4V titanium alloy at 400°C, 500°C, 600°C, 700°C and 800°C, respectively.

The partial EDS diagrams of the wear scar after the friction experiment at different temperatures are shown in figure 9. As shown in figure 9*a–e*, four major elements including Ti, C, O and Si are all detected at the wear scar. Figure 9*a,b* shows the EDS images of the wear scar at 400°C and 500°C, respectively. It can be clearly observed that the titanium alloy discs have deep grooves filled with small abrasive chips. These wear debris are a mixture of titanium alloy abrasive particles and MLG/SiO_2_ nanocomposites. Figure 9*c* is the EDS image of the wear scar at 600°C. Due to the residue of lubricant, there are no obvious furrows on the surface of titanium alloy. Figure 9*e,f* is the EDS image of the wear scar at 700°C and 800°C. Deeper furrows could be observed and some abrasive chips with larger particles existed on the surface of titanium alloy. Elemental analysis of the abrasive chips was carried out, which were mainly dominated by the abrasive particles of titanium alloy.

**Figure 10.**
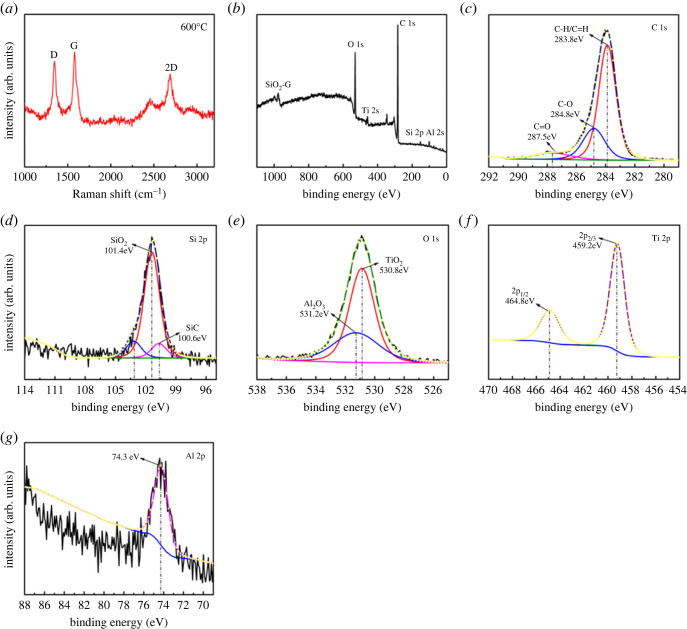
(*a*) Raman spectra (*b–g*) the XPS spectra of wear scar of Ti6Al4V titanium alloy at 600°C.

Combined with the above results and analysis, the COF is minimum at 600°C. SEM and EDS analyses of the wear scar of the titanium alloy disc at 600°C reveal the presence of lubricant residues. Therefore, the low COF may be due to the presence of MLG/SiO_2_ composite. As shown in figure 10*a–g*, the wear scar was analysed by Raman spectroscopy and XPS. It was determined whether the chemical reaction occurred during the friction process and whether MLG/SiO_2_ composites were present at the wear scar. In the Raman image (figure 10*a*), it can be observed that the peaks mainly appear at 1346 cm^−1^, 1577 cm^−1^ and 2691 cm^−1^, which are the D-band caused by MLG disorder, G-band and the second-order two-dimensional band of MLG, respectively. Therefore, Raman spectroscopy indicated the MLG existing in the wear scar at 600°C [53,54].

Figure 10*b* shows the full spectrum at the wear scar on the disc after the high-temperature friction test at 600°C. The elements of C 1s, O 1s, Si 2p, Ti 2p, Al 2p and SiO_2_-G were presented [55]. Three individual C 1s peaks were centred at 283.8, 284.88 and 287.5 eV, and which are related to C-H/*C*=H, C-O and *C*=O, as shown in figure 10*c*. As shown in figure 10*d*, the two peaks located at 100.6 and 101.4 eV correspond to SiO_2_ and SiC, respectively. The existence of SiC may be due to the phase change of the SiO_2_ nanoparticles in the lubricant after long-term high-temperature friction experiments [47]. Figure 10*c,d* illustrates that after the high-temperature frictional test, lubricant residue was found on the surface of the wear scar. The O 1s peak and the Al 2p peak appeared at 531.2 and 74.7 eV in figure 10*g*, indicating the presence of Al_2_O_3_. The O 1s peak at 530.8 eV is attributed to O^2−^ in the TiO_2_ lattice. As shown in figure 10*f*, Ti 2p XPS spectrum shows double peaks at 458.5 and 464.2 eV, which corresponds to the Ti 2p 3/2 and Ti 2p 1/2 characteristic peaks [56,57]. Raman images and XPS spectra indicate that there is residual of MLG/SiO_2_ composites in the wear scar. Due to the long time under high temperature and load, the friction chemical reaction occurred on the wear surface, forming a film composed of Al_2_O_3_, TiO_2_ and a small amount of residual lubricant.

### 3.4. Lubrication mechanism of the coating

**Figure 11.**
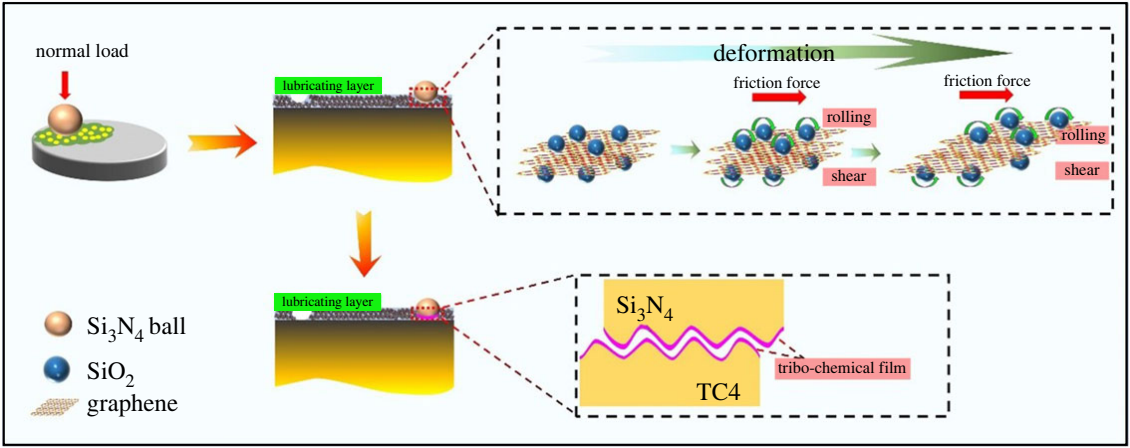
Schematic illustration of the lubrication model of the solid lubricant coating.

Based on the observed results and the wear scar analysis, the lubrication mechanisms of the MLG/SiO_2_ composites solid lubricant coatings are proposed, as shown in figure 11. During the frictional experiment, there are two frictional stages. At the initial stage of the friction experiment, the lubricating coating existed between the Si_3_N_4_ ball and the titanium alloy disc, which acted as a buffer layer and avoids the direct contact between the ball and the disc. Under the action of frictional shear force, the hard SiO_2_ nanoparticles in the composites carried severe rubbing stress and acted as ball bearings to frictional forces. In addition, SiO_2_ nanoparticles had a polishing effect on the friction surface. Meanwhile, relative sliding occurred between the layers of MLG and reduced shear resistance achieving less friction. Since the synergistic effect of MLG and SiO_2_, the solid lubricants showed excellent high-temperature lubricating performance. The lubrication mechanism at this stage is a mixture of sliding friction and rolling friction, and finally achieves the goal of reducing friction [16,58,59]. With the extension of frictional time, a large amount of the lubricating coating was consumed and a small part of the lubricant was deposited on the friction interface, the exposed part of matrix was oxidized at the same time. Under the action of large loads, the tightly bound lubricating film including the residual lubricant of Al_2_O_3_ and TiO_2_ was formed. A tribo-chemical film of metal oxide is generated on the surface of the friction pairs that is the key to reduce the COF. Due to the existence of frictional shear stress, a portion of the metal oxide was transferred into the surface of the Si_3_N_4_ ball, forming a frictional contact from the metal oxide to metal oxide and avoiding direct contact between the friction pairs [60].

## 4. Conclusion

The MLG/SiO_2_ composites were successfully fabricated by sol–gel method and presented excellent oxidation resistance at high temperature. The eco-friendly solid lubricant coatings were fabricated by mixing the MLG/SiO_2_ composites and NaPO_3_, having the good lubrication properties at high temperature. By increasing the friction temperature, the COF at 600°C was the lowest and compared with the COF at 800°C decreased 15%. It is noteworthy that the wear rate of the upper specimen (Si_3_N_4_ balls) significantly increased, it is due to the softening and plastic deformation of the titanium alloy and the coating at high temperature. The friction-reducing mechanism of the solid coatings was closely related to the lamellar structure of MLG and the structure of SiO_2_ ball. In addition, the tightly bound lubricating film generated on the wear scar, which avoided direct contact between friction pairs. In summary, these MLG/SiO_2_ composite-based solid lubricants have a great potential, which has a guiding value for designing the eco-friendly high-temperature solid lubricant.

## Data Availability

The raw data are available at the Dryad Digital Repository: https://doi.org/10.5061/dryad.p2ngf1vt6 [61].

## Permission to carry out fieldwork

The appropriate permissions and licences were not needed in this investigation.
